# Nature nurtures our wellbeing: primary emotions and attachment mediate our psychological adjustment via connectedness to nature

**DOI:** 10.3389/fcogn.2026.1857956

**Published:** 2026-05-29

**Authors:** Amadeu Quelhas Martins, Júlia Fonte, Nuno Torres, Paulo Ferrajão

**Affiliations:** 1 Research Unit in Design and Communication, UNIDCOM/IADE, Lisbon, Portugal; 2 Research Centre in Sports and Human Development, Universidade Europeia, Lisbon, Portugal; 3 Centro de Investigacao em Desporto Saude e Desenvolvimento Humano, Vila Real, Portugal; 4 Instituto Universitario de Ciencias Psicologicas Sociais e da Vida, Lisbon, Portugal

**Keywords:** attachment patterns, nature connectedness, primary emotional systems, psychological symptoms, structural equation modeling

## Abstract

**Introduction:**

Research suggests that the psychological benefits of natural environments are not uniformly experienced, with individual differences in nature connectedness potentially shaped by temperament traits and attachment patterns moderating their impact on psychological wellbeing. Drawing on Affective Neuroscience theory, which conceptualizes basic human motivational tendencies as primary emotional systems (CARE, SEEKING, PLAY, FEAR, ANGER, PANIC), we examined whether these systems are linked to anxiety and depression symptoms through attachment patterns and nature connectedness.

**Methods:**

A non-clinical sample of 592 Portuguese adults, aged 18 or older, completed self-report measures of primary emotional systems, attachment, nature connectedness, and psychological symptoms. A multiple step mediation was tested through Structural Equation Modeling, employing bootstrapped confidence intervals to test for indirect effects.

**Results:**

Four association pathways emerged from the data, namely (1) a Biophilic Connection path, in which positive emotional systems were associated with lower anxiety and depression via greater connectedness to nature; (2) an Attachment to Nature path, whereby secure attachment tendencies supported wellbeing through enhanced nature connectedness; (3) an Anger-Nature Disconnection path, in which anger-related tendencies were linked to poorer outcomes through reduced connection to nature; and (4) a Fear-Nature Disconnection path, whereby fear-related emotions and attachment insecurity contributed to worse mental health via diminished nature connectedness.

**Discussion:**

Our findings highlight distinct emotional and relational trajectories through which connectedness to nature may influence psychological wellbeing, offering an integrative framework for understanding individual variability in nature's psychological benefits.

## 1 Introduction

### 1.1 Individual variability in nature-related outcomes

A substantial body of empirical research consistently demonstrates that spending time in nature—like parks and green spaces—improves individuals' mental health and wellbeing (Bratman et al., 2015; Chang et al., 2024; Patwary et al., 2024). However, these benefits are not uniformly experienced across individuals (Pouso et al., 2021). One key factor underlying this variability is individuals' sense of connection to the natural world, which research shows significantly shapes the benefits gained from nature (Chang et al., 2024).

Connection to nature is conceptualized as a relatively stable psychological disposition, characterized by a perceived sense of oneness with the natural world and encompassing both affective and cognitive dimensions of identification with nature (Mayer and Frantz, 2004; Schultz and Tabanico, 2007). Individuals with a stronger connection to nature tend to report greater reductions in stress, anxiety, and depression following exposure to natural environments (Chang et al., 2020, 2024). Despite growing interest in this construct, research examining the mechanisms underlying individual differences in nature connectedness—and how these influence mental health outcomes—remains limited.

Research on how individual differences in nature connection influence the link between nature contact and psychological wellbeing remains limited. These differences may reflect genetic and environmental factors (Chang et al., 2022). Innate primary emotional systems, as described by Affective Neuroscience (AN), offer a framework for understanding such variability, representing genetically rooted predispositions that shape emotional responses and behavior across mammals (Davis and Montag, 2019; Ferrajão et al., 2024; Pihkala, 2020).

### 1.2 Primary emotional systems as temperamental traits

Seven core emotional systems—SEEKING, CARE, LUST, PLAY, ANGER, FEAR, and PANIC—have been identified as *evolutionarily conserved neural circuits*, which guide behavior and cognition (Davis and Panksepp, 2018; Panksepp, 1998). SEEKING, ANGER, and FEAR emotional outputs are fundamentally associated with instinctive behavioral responses and primarily mediated by the brain's phylogenetically older structures, often designated as the "reptilian brain,” whereas CARE, LUST, PANIC, and PLAY are social emotions predominantly mediated by the limbic system, a neural network essential for sophisticated emotional and social functioning (Panksepp, 1998).

A measure was developed to assess individual differences in six primary emotional systems (SEEKING, CARE, PLAY, ANGER, FEAR, and PANIC), the Affective Neuroscience Personality Scales (ANPS; Davis et al., 2003) was developed to assess individual differences across six primary emotional systems. The measure does not include the LUST system, as the incorporation of sexuality-related items was considered likely to introduce bias in the assessment of the remaining emotional systems, and this dimension has not been consistently identified in studies employing other personality frameworks (Montag et al., 2021). As such, due to those conceptual, cultural, and methodological challenges in studying LUST (Davis and Montag, 2019), this study will focus only on the six remaining primary emotional systems.

Primary emotional systems can be grouped into positive or negative types according to the affective states they generate and the adaptive survival functions they serve (Panksepp, 1998). Positive emotional systems promote emotional arousal associated with approach-oriented behaviors. The CARE system promotes nurturing behaviors toward family and close companions by motivating protection, comfort, and support; the PLAY system facilitates social bonding through enjoyable, physically interactive experiences that generate positive emotions and strengthen relationships; and the SEEKING system drives curiosity and exploration, fostering the pursuit of novel experiences and goals while reinforcing purposeful behavior (Panksepp, 1998; Panksepp and Biven, 2012).

Conversely, negative primary emotional systems are activated to suppress or mitigate unpleasant stimuli. The FEAR system is engaged to protect against threats, producing anxiety, worry, and physiological and psychological tension; the ANGER system responds to challenges to vital resources or physical restraint, eliciting irritation and frustration; and the PANIC system is activated by social separation, particularly the loss of an attachment figure, resulting in feelings of loneliness and grief (Montag and Davis, 2018).

According to AN theory, primary emotional systems comprise temperamental personality traits that regulate and shape how individuals perceive and respond to their environment (Panksepp and Biven, 2012). Importantly, empirical evidence links these systems to psychopathology, particularly anxiety and depression. Elevated PANIC and FEAR, together with reduced PLAY and SEEKING, have been consistently associated with higher levels of anxiety and depressive symptoms (Fuchshuber et al., 2019a; Montag and Panksepp, 2017).

### 1.3 Primary emotional systems and connection to nature

Wilson's (1984) Biophilia Hypothesis proposes that humans exhibit an intrinsic biological tendency to connect emotionally with nature, reflecting evolutionary adaptations that support both survival and mental wellbeing. While empirical research linking primary emotional systems to nature connectedness is still emerging, personality traits-associated with primary emotional systems-play a distinctive role in determining one's connection to the natural environment (Marengo et al., 2021; Panksepp, 2011). Therefore, individual differences in nature connectedness may, at least partially, reflect underlying emotional predispositions.

Positive emotional systems appear to play a crucial role in enhancing individuals' connectedness to nature. Within the framework linking personality trait factors and affective-neuroscience systems, Agreeableness, and Openness to Experience have been respectively associated with the PLAY, CARE, and SEEKING systems (Marengo et al., 2021; Montag and Panksepp, 2017). Extraversion has been shown to predict stronger affective bonds with nature, reflecting the influence of the PLAY system in fostering joy and social engagement with the natural world (Nisbet et al., 2009; Tam, 2013). Similarly, Agreeableness correlates positively with connectedness to nature, suggesting the CARE system's contribution to compassionate orientations toward the environment (Di Fabio and Rosen, 2019). Finally, individuals high in Openness to Experience tend to report greater connectedness to nature, consistent with the SEEKING system's drive for curiosity and exploration within natural settings (Lee et al., 2015).

Negative primary emotional systems influence the human–nature relationship in complex ways. Research links the Big Five trait of Neuroticism to the FEAR and PANIC systems (Davis and Panksepp, 2011; Marengo et al., 2021), yet large-scale studies indicate only a weak, context-dependent association between Neuroticism and nature connectedness (Lee et al., 2015; Nisbet et al., 2009; Tam, 2013; Wu, 2025), suggesting indirect rather than direct effects. Similarly, higher ANGER is generally associated with lower Agreeableness (Marengo et al., 2021), implying a negative relationship with connectedness to nature (Di Fabio and Kenny, 2021; Di Fabio and Rosen, 2019). No study has yet examined primary emotional systems, nature connectedness, and psychological symptoms within a unified framework.

### 1.4 Attachment patterns and connection to nature

Beyond biological predispositions, proximal social environmental factors, particularly early interpersonal bonds, may contribute significantly to individual differences in nature relatedness (Chang et al., 2022; Pasca et al., 2022). Among these, attachment style emerges as a significant determinant of individuals' connectedness to nature (Shorey et al., 2006). Rooted in attachment theory, early interactions with caregivers are internalized as internal working models (IWMs) that guide emotional regulation and relational patterns (Mikulincer and Shaver, 2016; Panksepp, 1998; Schore, 2001). Secure attachment arises from caregivers' consistent sensitivity and emotional availability, fostering emotional stability and resilience, whereas insecure attachment develops through experiences of unresponsiveness or insufficient support (Bowlby, 1973).

Secure attachment is generally associated with better mental health outcomes (Sroufe, 2005). In contrast, insecure attachment patterns have been consistently linked to both anxiety and depression symptoms in adolescents and adults (Brumariu and Kerns, 2010; Dagan et al., 2018; Marganska et al., 2013). These individuals may experience difficulties in forming stable and supportive relationships, which may further exacerbate psychological distress (Mikulincer et al., 2007).

There is growing evidence that primary emotional systems may act as anchoring mechanisms shaping personality in a bottom-up manner, while also underpinning attachment processes through similar underlying mechanisms (Freund et al., 2024; Panksepp, 1998). For instance, a prior study in a non-clinical adult sample showed that comfort with dependence was negatively associated with ANGER, FEAR, and PANIC, and positively associated with PLAY, SEEK, and CARE; comfort with closeness was positively related to PLAY, SEEK, and CARE; conversely, anxiety about being unloved was negatively associated with PLAY, SEEK, and CARE (Fuchshuber et al., 2019b).

Recent research has increasingly examined how primary emotional systems relate to psychological symptoms in non-clinical populations, particularly through the mediating role of attachment orientations. However, no study has yet explored the association between these emotional systems and symptoms of anxiety and depression via attachment patterns. Evidence indicates that negative primary emotional systems—especially PANIC—contribute to psychological distress mainly through heightened attachment anxiety (Freund et al., 2024; Fuchshuber et al., 2024; Roithmeier et al., 2024), while ANGER shows a positive and CARE a negative association with gaming disorder, both mediated by avoidant attachment (Wimmer et al., 2025).

Attachment orientations may also play a role in linking primary emotional systems to individuals' relationships with the natural environment. Although empirical evidence remains limited, research on related constructs indicates that early interpersonal bonds shape how people relate to nature. For instance, Li et al. (2021) found that individuals characterized by strong autonomy and self-reliance, alongside a strong motivation to form and maintain social connections, displayed lower levels of nature connectedness, suggesting a compensatory function of nature in addressing unmet attachment needs. Secure attachment, by fostering safety and curiosity, may encourage stable and meaningful connections with the natural world (Weinstein et al., 2009), facilitating deeper emotional bonds that extend beyond human relationships (Lumber et al., 2017). Over time, such connections can develop into an attachment to nature (Jordan, 2009), where the environment functions as a secure base offering comfort, stability, and emotional resilience (Little and Derr, 2018).

Despite advances in these areas, no study has yet integrated primary emotional systems, attachment patterns, and nature connectedness within a unified framework. Drawing on the Biophilia Hypothesis (Wilson, 1984), the present study proposes that biologically grounded emotional systems and attachment orientations jointly shape individuals' connectedness to nature and psychological wellbeing. Specifically, positive emotional systems (e.g., SEEKING, CARE, and PLAY) may foster engagement with nature, whereas negative systems (e.g., FEAR, PANIC, and ANGER) may hinder it, particularly in the context of insecure attachment (Fuchshuber et al., 2019b; Mikulincer and Shaver, 2016; Panksepp, 1998). This framework suggests a sequential pathway linking primary emotional systems, attachment orientations, nature connectedness, and symptoms of anxiety and depression. The present study seeks to advance understanding of the mechanisms linking emotional predispositions, human–nature relationships, and mental health outcomes.

### 1.5 Purpose of the study

To the best of our knowledge, no previous research has systematically investigated the interaction between innate primary emotional predispositions and environmental factors in shaping nature connectedness, nor examined how this interaction relates to psychological symptomatology. A clearer understanding of these associations is crucial for informing the development of targeted nature-based mental health interventions. The present study aimed to elucidate the emotional mechanisms linking nature connectedness to symptoms of anxiety and depression by analyzing the associations among heritable emotional traits, attachment patterns, and connection to nature in a non-clinical sample of Portuguese adults.

The following hypotheses were advanced: (H1) Higher PANIC and FEAR levels will be directly associated with increased anxiety and depressive symptoms; (H2) Higher PLAY and SEEKING levels will also be directly associated with decreased anxiety and depressive symptoms; (H3) PANIC and FEAR will indirectly predict anxiety and depressive symptoms via greater attachment anxiety; (H4) CARE, PLAY, and SEEKING will indirectly predict lower anxiety and depressive symptoms through enhanced connectedness to nature; (H5) ANGER will indirectly predict higher anxiety and depressive symptoms through reduced connectedness to nature; (H6) In a serial mediation model: (H6a) CARE, SEEKING, and PLAY will increase attachment closeness and dependence, enhancing connectedness to nature and reducing anxiety and depressive symptoms; (H6b) ANGER will decrease attachment closeness and dependence, reducing connectedness to nature and increasing anxiety and depressive symptoms; (H6c) PANIC and FEAR will increase attachment anxiety, lowering connectedness to nature and raising anxiety and depressive symptoms.

## 2 Method

### 2.1 Participants

592 Portuguese adults (*M* = 36.13 years, SD = 16.32; range = 18–83) agreed to participate. Women were overrepresented in this sample. Most participants were single, whereas smaller proportions were married, cohabiting, divorced, or widowed. Approximately half had completed higher education, over 40% had completed secondary education, and the remainder had nine or fewer years of formal schooling. About half were employed, nearly 40% were students, and the rest were unemployed or retired. The majority belonged to the middle socioeconomic class, with a few in the upper tier (Table 1).

**Table 1:** Sample demographic characteristics.

Variable	*n*	%
Sex	
Female	369	62.3
Male	223	37.7
Civil status	
Single	304	51.4
Married or cohabitation	229	38.7
Divorced	50	8.4
Widowed	9	1.5
Education	
Less than 9 years of education	13	2.2
9 years of education	27	4.6
12 years of education	253	42.7
Higher education	299	50.5
Professional status	
Student	220	37.2
Employed	297	50.2
Unemployed	40	6.8
Retired	35	5.9
Socioeconomic status	
Low	58	9.8
Medium	433	73.1
Medium-high	94	15.9
High	7	1.2

### 2.2 Procedure

This study is part of a broader project exploring the relationship between individuals and nature, and its association with mental health among Portuguese participants. Ethical approval was granted by the Institutional Review Board of Universidade Europeia. Participants were recruited through convenience sampling via online invitations shared through mailing lists and social media (Facebook, Instagram, LinkedIn, WhatsApp). Informed consent was obtained electronically before completing the questionnaire. Following the ethical principles of the Declaration of Helsinki, participants were fully informed about the study's aims and procedures. Participation was voluntary, with no financial compensation provided.

Data was gathered via an online survey comprising a series of self-report instruments that were edited into Microsoft Forms^®^. Participants were invited to complete a battery of questionnaires designed to assess primary emotional systems, attachment patterns, connection to nature, anxiety and depressive symptoms, and sociodemographic characteristics. Access to the survey was provided through a link, and the completion time for the entire set of measures was approximately 20 min.

### 2.3 Measures

#### 2.3.1 . Primary emotional systems

The Brief Form of the Affective Neuroscience Personality Scales (BANPS; Barrett et al., 2013; Gurfinkel et al., 2019) is a 33-item self-report instrument designed to assess six primary emotional systems underlying affective behavior: ANGER, FEAR, PANIC, PLAY, SEEKING, and CARE. Items are rated on a five-point Likert scale (1 = strongly disagree to 5 = strongly agree), with subscale scores calculated by summing relevant items. The subscales demonstrated acceptable to good internal consistency, with Cronbach's alphas of 0.81 (ANGER), 0.79 (FEAR), 0.85 (PANIC), 0.83 (PLAY), 0.75 (SEEKING), and 0.78 (CARE).

#### 2.3.2 Attachment orientations

The Revised Adult Attachment Scale (RAAS; Collins and Read, 1990; Canavarro, 1997) is an 18-item self-report instrument using a 5-point Likert scale (1 = *Not at all characteristi*c, 5 = *very characteristic*). It assesses three attachment dimensions: closeness (“I find it relatively easy to get close to others”), dependence (“I am comfortable depending on others”), and anxiety (“I often worry that my partner does not really love me”). Internal consistency was satisfactory, with Cronbach's α = 0.78, 0.75, and 0.88 for closeness, dependence, and anxiety, respectively.

#### 2.3.3 Connectedness to nature

The Revised Environmental Identity Scale (Revised EID; Clayton et al., 2021; Ferrajão et al., 2024) is a 14-item self-report measure assessing the degree of environmental identity. Participants rate each item on a 7-point Likert scale (1 = *not at all true of me* to 7 = *completely true of me*). Cross-cultural validations, including Portuguese and Italian versions, revealed a two-factor structure encompassing Connectedness with Nature and Protection of Nature (Ariccio and Mosca, 2023; Ferrajão et al., 2024). The present study employed the Connectedness with Nature subscale, with scores computed by summing item responses. Internal consistency was excellent (α = 0.94).

#### 2.3.4 Psychological symptoms

The Brief Symptom Inventory-18 (BSI-18; Derogatis, 2001; Canavarro et al., 2016) is an 18-item self-report instrument assessing psychological distress in clinical and non-clinical populations. Items are rated on a 5-point Likert scale (0 = *not at all* to 4 = *extremely*), reflecting distress experienced over the past 7 days. The BSI-18 comprises three symptom dimensions: anxiety, depression, and somatization. The present study analyzed only anxiety and depressive symptoms, with total scores calculated by summing the respective items. Internal consistency was satisfactory, with Cronbach's α of 0.88 for Anxiety and 0.75–0.90 for Depression.

#### 2.3.5 Sociodemographic data

The sociodemographic questionnaire comprised items designed to gather information on participants' sex, age, marital status, educational attainment, employment status, and socioeconomic background.

### 2.4 Data analysis

Descriptive statistics-means, standard deviations, and minimum and maximum values-were computed for study variables. Bivariate relationships were examined using Pearson correlation analyses, with coefficients interpreted as strong (±0.50–1.00), moderate (±0.30–0.49), or weak (<±0.29). All analyses were conducted in IBM SPSS (Armonk, NY, USA) Statistics for Windows, Version 29. To test the hypothesized indirect effects of primary emotional systems on anxiety and depression symptoms via attachment patterns and connectedness to nature, a multiple-step mediation analysis with bootstrapped confidence intervals was performed (Hayes, 2013). The model was further evaluated using structural equation modeling (SEM). The dataset was free from missing values, as only participants who answered all questionnaire items were included.

This study examined both direct effects of primary emotional systems on anxiety and depressive symptoms and their indirect effects via attachment patterns and connectedness to nature. Specifically, a two-step mediation model was tested, in which primary emotional systems were linked to anxiety and depressive symptoms through attachment patterns and closeness to nature. All variables were treated as observed measures.

The model was evaluated using Structural Equation Modeling (SEM) in AMOS 29 with Maximum Likelihood estimation, following Hoyle and Smith (1994). Model fit was assessed via conventional SEM criteria: (a) non-significant chi-square (χ^2^) test; (b) values for the Comparative Fit Index (CFI), Normed Fit Index (NFI), and Tucker-Lewis Index (TLI) exceeding 0.95; and (c) Root Mean Square Error of Approximation (RMSEA) and Standardized Root Mean Square Residual (SRMR) values below 0.08. Indirect effects were assessed using the bootstrapping procedure of Preacher and Preacher and Hayes (2008) with 5,000 resamples. Bias-corrected and accelerated 95% confidence intervals (CIs) were computed for single- and two-step mediation effects. Effects were deemed significant when the CI did not include zero.

## 3 Results

### 3.1 Intercorrelations between study variables

PLAY was moderately positively associated with CARE and attachment closeness, and weakly positively associated with SEEKING, attachment dependence, and Connectedness to nature, while showing weak negative associations with PANIC, attachment anxiety, and depressive symptoms. SEEKING was weakly positively associated with CARE, attachment closeness, and Connectedness to nature, and weakly negatively associated with FEAR and anxiety symptoms. CARE was strongly positively associated with attachment closeness, weakly positively associated with attachment dependence and Connectedness to nature, and weakly negatively associated with ANGER, FEAR, and depressive symptoms. ANGER showed moderate positive associations with PANIC, weak positive associations with FEAR, attachment anxiety, Connectedness to nature, and both anxiety and depressive symptoms, and weak negative associations with attachment closeness and dependence. PANIC was strongly positively associated with FEAR, attachment anxiety, and both anxiety and depressive symptoms, moderately negatively associated with attachment closeness and dependence, and weakly negatively associated with Connectedness to nature. FEAR exhibited strong positive associations with anxiety symptoms, moderate positive associations with attachment anxiety and depressive symptoms, and a weak negative association with attachment dependence. Attachment closeness was moderately positively associated with attachment dependence, weakly positively associated with Connectedness to nature, moderately negatively associated with attachment anxiety, and weakly negatively associated with anxiety and depressive symptoms. Attachment dependence was moderately negatively associated with attachment anxiety and depressive symptoms, and weakly negatively associated with anxiety symptoms. Anxiety and depressive symptoms were strongly positively associated. All other associations were non-significant (Table 2).

**Table 2:** Correlation matrix and descriptive statistics of study variables.

Variables	1	2	3	4	5	6	7	8	9	10	11	12
1. PLAY	–	0.23^***^	0.44^***^	−0.07	−0.20^***^	0.07	0.43^***^	0.22^***^	−0.12^**^	0.09^*^	−0.06	−0.12^**^
2. SEEKING		–	0.22^***^	−0.07	−0.03	−0.13^***^	0.17^***^	0.04	−0.02	0.21^***^	−0.11^*^	−0.03
3. CARE			–	−0.16^***^	−0.08	−0.15^***^	0.50^***^	0.22^***^	−0.06	0.16^**^+	0.02	−0.10^*^
4. ANGER				–	0.40^***^	0.29	−0.26^***^	−0.21^***^	0.29^***^	0.17^***^	0.29^***^	0.30^***^
5. PANIC					–	0.56^***^	−0.33^***^	−0.34^***^	0.58^***^	−0.12^**^	0.58^***^	0.69^***^
6. FEAR						–	−0.08	−0.22^***^	0.45^***^	−0.05	0.51^***^	0.45^***^
7. Attachment closeness							–	0.36^***^	−0.36^***^	0.11^*^	−0.23^***^	−0.30^***^
8. Attachment dependence								–	−0.41^***^	−0.07	−0.27^***^	−0.34^***^
9. Attachment anxiety									–	−0.01	0.50^***^	0.58^***^
10. Connectedness to nature										–	−0.03	−0.05
11. Anxiety symptoms											–	0.78^***^
12. Depression symptoms												–
Descriptive statistics	
Mean	22.56	21.85	15.11	16.79	17.58	17.59	2.37	3.17	2.67	53.67	7.55	7.08
Standard Deviation	4.28	3.61	2.87	4.39	5.20	3.49	0.66	0.69	1.08	14.25	5.93	6.27
Minimum	8	9	5	6	6	7	1.00	1.33	1.00	10	0	0
Maximum	30	30	20	30	30	25	4.33	5.00	5.00	70	24	24

^*^p < 0.05, ^**^p < 0.01, ^***^p < 0.001.

### 3.2 Serial mediation analyses

**Figure 1:**
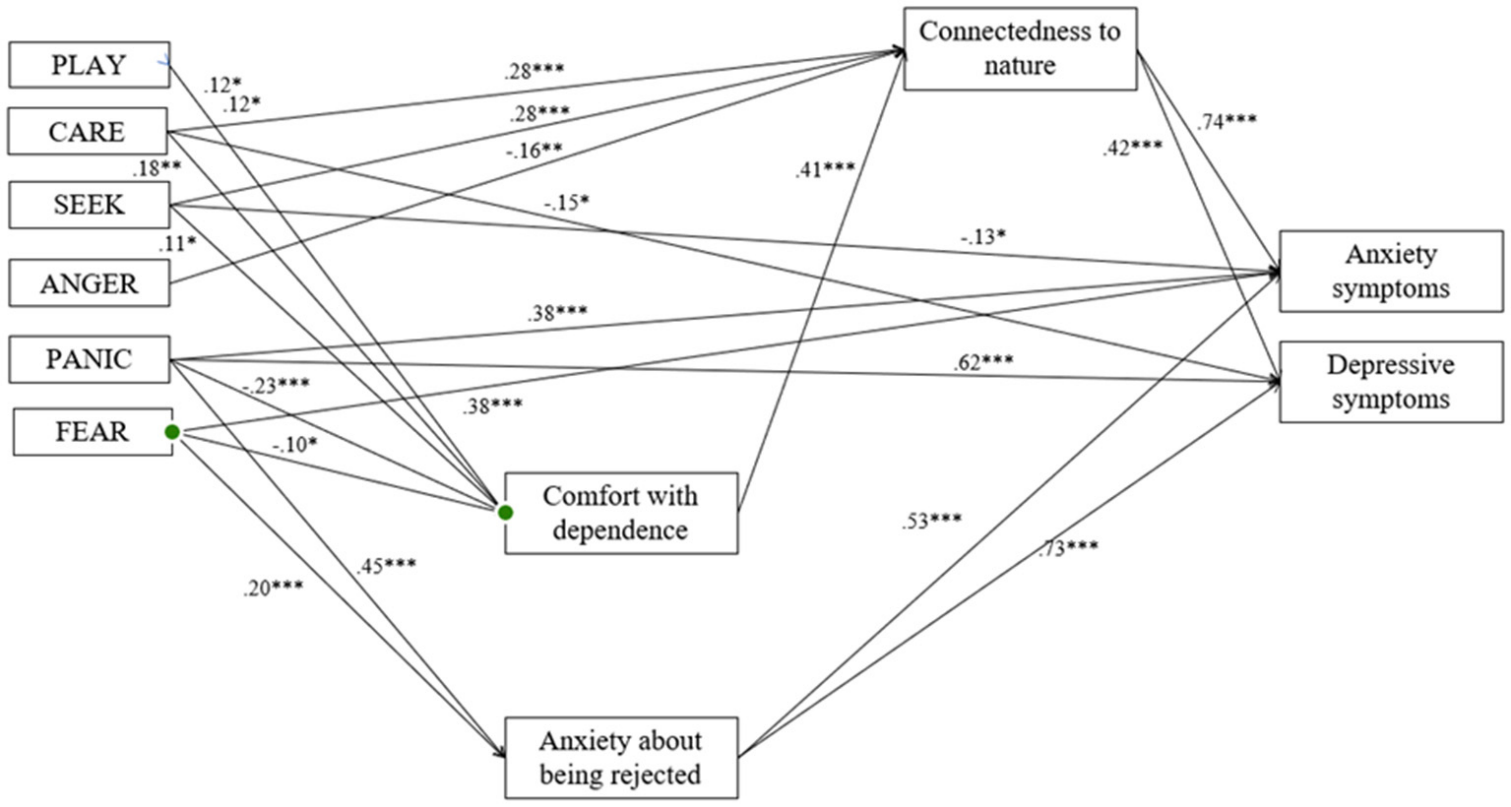
A serial mediational integrated model for anxiety and depression symptoms by primary emotional systems through attachment orientations. Rectangles indicate measured variables. Unidirectional arrows depict hypothesized directional links. Standardized maximum likelihood parameters are used. Bold line estimates are statistically significant paths. *N* = 592; *p* < 0.05, *p* < 0.01, *p* < 0.001.

A two-step mediation model was tested in which primary emotional systems were indirectly associated with anxiety and depressive symptoms via attachment patterns and connectedness to nature. The model demonstrated excellent fit (χ^2^(9) = 14.13, *p* = 0.12; NFI = 0.99; CFI = 0.99; TLI = 0.99; RMSEA = 0.03; SRMR = 0.02), with standardized results shown in Figure 1. Direct effects indicated that lower SEEK (*b* = −0.13, *p* < 0.05) and higher PANIC (*b* = 0.38, *p* < 0.001) and FEAR (*b* = 0.38, *p* < 0.001) were associated with elevated anxiety symptoms (Table 3). Additionally, lower CARE (*b* = −0.15, *p* < 0.05) and higher PANIC (*b* = 0.62, *p* < 0.001) were linked to higher depressive symptoms (Table 4).

**Table 3:** Bootstrapped point estimate for direct and indirect effects and 95% confidence intervals for predicting anxiety symptoms by primary emotional systems through attachment orientations.

Path	Point estimate	SE	BCa 95% CI (lower, upper)
Direct effect of PLAY	−0.01	0.05	(−0.11, 0.09)
Direct effect of CARE	−0.11	0.08	(−0.27, 0.05)
Direct effect of SEEK	−0.15	0.05	(−0.25, −0.05)^**^
Direct effect of ANGER	0.06	0.05	(−0.04, 0.16)
Direct effect of PANIC	0.37	0.05	(0.27, 0.47)^***^
Direct effect of FEAR	0.37	0.07	(0.23, 0.51)^***^
Indirect effect of PLAY through comfort with closeness	−0.03	0.03	(−0.09, 0.03)
Indirect effect of PLAY through comfort with dependence	−0.02	0.04	(−0.09, 0.05)
Indirect effect of PLAY through anxiety about being rejected	0.01	0.05	(−0.09, 0.11)
Indirect effect of CARE through comfort with closeness	−0.08	0.06	(−0.20, 0.04)
Indirect effect of CARE through comfort with dependence	−0.04	0.03	(−0.10, 0.02)
Indirect effect of CARE through anxiety about being rejected	−0.01	0.01	(−0.03, 0.01)
Indirect effect of SEEK through comfort with closeness	−0.01	0.02	(−0.04, 0.02)
Indirect effect of SEEK through comfort with dependence	−0.02	0.02	(−0.05, 0.02)
Indirect effect of SEEK through anxiety about being rejected	0.01	0.01	(−0.01, 0.03)
Indirect effect of ANGER through comfort with closeness	0.02	0.02	(−0.01, 0.05)
Indirect effect of ANGER through comfort with dependence	0.01	0.03	(−0.05, 0.07)
Indirect effect of ANGER through anxiety about being rejected	0.01	0.02	(−0.03, 0.05)
Indirect effect of PANIC through comfort with closeness	0.03	0.04	(−0.05, 0.11)
Indirect effect of PANIC through comfort with dependence	0.03	0.05	(−0.06, 0.12)
Indirect effect of PANIC through anxiety about being rejected	0.28	0.03	(0.22, 0.34)^***^
Indirect effect of FEAR through comfort with closeness	0.01	0.04	(−07, 0.09)
Indirect effect of FEAR through comfort with dependence	0.02	0.06	(−0.10, 0.12)
Indirect effect of FEAR through anxiety about being rejected	0.32	0.04	(0.25, 0.39)^***^

BCa, bias corrected and accelerated; CI, confidence intervals; Confidence intervals that do not include 0 (null association) are significant. ^**^p < 0.01, ^***^p < 0.001.

**Table 4:** Bootstrapped point estimate for direct and indirect effects and 95% confidence intervals for predicting depression symptoms by primary emotional systems through attachment orientations.

Path	Point estimate	SE	BCa 95% CI (lower, upper)
Direct effect of PLAY	−0.07	0.05	(−0.17, 0.03)
Direct effect of CARE	−0.15	0.07	(−0.28, −0.02)^*^
Direct effect of SEEK	−0.08	0.05	(−0.18, 0.02)
Direct effect of ANGER	0.02	0.05	(−0.07, 0.11)
Direct effect of PANIC	0.62	0.05	(0.52, 0.72)^***^
Direct effect of FEAR	0.09	0.07	(−0.06, 0.22)
Indirect effect of PLAY through comfort with closeness	−0.06	0.05	(−0.16, 0.04)
Indirect effect of PLAY through comfort with dependence	−0.01	0.03	(−0.07, 0.05)
Indirect effect of PLAY through anxiety about being rejected	−0.03	0.06	(−0.14, 0.17)
Indirect effect of CARE through comfort with closeness	−0.05	0.04	(−0.03, 0.13)
Indirect effect of CARE through comfort with dependence	−0.07	0.08	(−0.22, 0.22)
Indirect effect of CARE through anxiety about being rejected	−0.02	0.02	(−0.05, 0.01)
Indirect effect of SEEK through comfort with closeness	−0.04	0.03	(−0.10, 0.02)
Indirect effect of SEEK through comfort with dependence	−0.03	0.02	(−0.07, 0.01)
Indirect effect of SEEK through anxiety about being rejected	0.01	0.02	(−0.03, 0.05)
Indirect effect of ANGER through comfort with closeness	0.05	0.04	(−0.02, 0.12)
Indirect effect of ANGER through comfort with dependence	0.03	0.03	(−0.03, 0.09)
Indirect effect of ANGER through anxiety about being rejected	0.01	0.03	(−0.04, 0.05)
Indirect effect of PANIC through comfort with closeness	0.02	0.03	(−0.04, 0.08)
Indirect effect of PANIC through comfort with dependence	0.08	0.05	(−0.01, 0.17)
Indirect effect of PANIC through anxiety about being rejected	0.45	0.06	(0.34, 0.56)^***^
Indirect effect of FEAR through comfort with closeness	0.03	0.04	(−04, 0.10)
Indirect effect of FEAR through comfort with dependence	0.01	0.04	(−0.06, 0.08)
Indirect effect of FEAR through anxiety about being rejected	0.20	0.04	(0.12, 0.28)^**^

BCa, bias corrected and accelerated; CI, confidence intervals; Confidence intervals that do not include 0 (null association) are significant. ^**^p < 0.01, ^***^p < 0.001.

Indirect effects via attachment orientations revealed that PANIC (*b* = 0.45, *p* < 0.001, 95% CI: 33, 0.57) and FEAR (*b* = 0.17, *p* < 0.01, 95% CI: 12, 0.22) were linked to anxiety symptoms through attachment anxiety (Table 3). Similarly, PANIC (*b* = 0.48, *p* < 0.001, 95% CI: 38, 0.58) and FEAR (*b* = 0.19, *p* < 0.01, 95% CI: 14, 0.24) were linked to depressive symptoms via the same pathway (Table 4). Higher PANIC and FEAR were associated with greater anxiety about being unloved, which subsequently related to higher anxiety and depressive symptoms (Tables 3, 4).

Indirect effects via connectedness to nature revealed that SEEKING (*b* = −0.26, *p* < 0.001, 95% CI: −0.36, −0.26), CARE (*b* = −0.28, *p* < 0.001, 95% CI: −0.38, −0.18), and ANGER (*b* = 0.13, *p* < 0.05, 95% CI: 0.07, 0.19) were linked to anxiety symptoms (Table 5). Also, SEEKING (*b* = −0.23, *p* < 0.001, 95% CI: −0.28, −0.18), CARE (*b* = −0.18, *p* < 0.01, 95% CI: −0.26, −0.10), and ANGER (*b* = 0.11, *p* < 0.01, 95% CI: 0.04, 0.18) were linked to depressive symptoms via the same pathway (Table 5). Specifically, higher SEEKING and CARE and lower ANGER were linked to greater connectedness to nature, which, in turn, was linked to reduced anxiety and depression (Table 5).

**Table 5:** Bootstrapped point estimate for direct and indirect effects and 95% confidence intervals for predicting anxiety and depressions symptoms by primary emotional systems through connectedness to nature.

Path	Point estimate	SE	BCa 95% CI (lower, upper)
Anxiety symptoms	
Direct effect of PLAY	−0.02	0.05	(−0.12, 0.08)
Direct effect of CARE	−0.01	0.08	(−0.16, 0.14)
Direct effect of SEEK	−0.12	0.05	(−0.22, −0.02)^*^
Direct effect of ANGER	0.09	0.05	(−0.01, 0.19)
Direct effect of PANIC	0.49	0.05	(0.40, 0.58)^***^
Direct effect of FEAR	0.43	0.07	(0.30, 0.56)^***^
Indirect effect of PLAY through connectedness to nature	−0.03	0.06	(−0.09, 0.15)
Indirect effect of CARE through connectedness to nature	−0.14	0.03	(−0.20, −0.08)^*^
Indirect effect of SEEK through connectedness to nature	−0.30	0.07	(−0.43, −0.17)^**^
Indirect effect of ANGER through connectedness to nature	0.15	0.06	(0.03, 0.27)
Indirect effect of PANIC through connectedness to nature	0.05	0.06	(−0.07, 0.17)
Indirect effect of FEAR through connectedness to nature	0.06	0.09	(−0.11, 0.23)
Depression symptoms	
Direct effect of PLAY	0.05	0.05	(−0.05, 0.15)
Direct effect of CARE	−0.21	0.07	(−0.35, −0.07)^**^
Direct effect of SEEK	−0.07	0.06	(−0.18, 0.04)
Direct effect of ANGER	0.01	0.05	(−0.09, 0.11)
Direct effect of PANIC	0.77	0.05	(0.68, 0.86)^***^
Direct effect of FEAR	0.19	0.07	(0.06, 0.32)^*^
Indirect effect of PLAY through connectedness to nature	−0.02	0.04	(−0.10, 0.06)
Indirect effect of CARE through connectedness to nature	−0.24	0.05	(−0.42, −0.06)^**^
Indirect effect of SEEK through connectedness to nature	−0.18	0.06	(−0.29, −0.07)^*^
Indirect effect of ANGER through connectedness to nature	0.12	0.05	(0.02, 0.22)^*^
Indirect effect of PANIC through connectedness to nature	0.03	0.03	(−0.03, 0.09)
Indirect effect of FEAR through connectedness to nature	0.04	0.05	(−0.06, 0.14)

BCa, bias corrected and accelerated; CI, confidence intervals; Confidence intervals that do not include 0 (null association) are significant. ^**^p < 0.01, ^***^p < 0.001.

As shown in Figure 1, only the significant paths are presented. The two-step indirect effects indicated that higher PLAY, SEEK, and CARE were linked to greater attachment dependence, which in turn were linked to stronger connectedness to nature, ultimately associated with lower anxiety and depressive symptoms. Conversely, higher PANIC and FEAR were associated with lower attachment dependence, leading to weaker connectedness to nature and higher anxiety and depressive symptoms. After excluding non-significant paths, the final model demonstrated excellent fit (χ^2^(11) = 14.36, *p* = 0.21; NFI = 0.99; CFI = 0.99; TLI = 0.99; RMSEA = 0.02; SRMR = 0.02).

## 4 Discussion

To the best of our knowledge, this study is the first to systematically examine the interplay between innate emotional predispositions and environmental relational factors—via primary emotional systems and attachment patterns—in shaping individuals' connectedness to nature. By integrating evolutionary, affective, and relational perspectives, the findings provide a more comprehensive understanding of how emotional predispositions and attachment orientations are associated with nature connectedness and psychological symptoms in a non-clinical adult sample. They also provide preliminary empirical support for four distinct pathways through which individual differences in nature connectedness may modulate the relationship between nature exposure and psychological wellbeing. The proposed hypotheses are discussed in detail.

The first hypothesis, which predicted that PANIC and FEAR would be directly associated with higher levels of anxiety and depressive symptoms, was only partially supported: FEAR was not directly linked to depressive symptoms. This finding may reflect a functional distinction between the two systems, as FEAR, characterized by acute threat detection and physiological hyperarousal, is more closely associated with anxiety (LeDoux, 2015), whereas PANIC is linked to separation distress, dysregulated affect, anhedonia, and hopelessness, which are core features of depression (Beck, 1976; Montag and Davis, 2018). The observed association between PANIC and depressive symptoms may also be attributable to the overlap between panic and depressive states, as panic attacks have been shown to predict subsequent depressive episodes (Kessler et al., 2005).

Our second hypothesis—that PLAY and SEEKING would be directly associated with higher levels of anxiety and depressive symptoms—was partially supported, as only SEEKING exhibited a direct relationship with anxiety symptoms. This result aligns with prior evidence indicating that reduced SEEKING activity, linked to motivational drive and exploratory behavior, increases vulnerability to anxiety (Davis and Montag, 2019; Panksepp, 1998). Individuals with a hypoactive SEEKING system may display reduced initiative, lower tolerance for uncertainty, and impaired stress regulation, factors associated with the onset of anxiety disorders (Davis and Montag, 2019).

The lack of a direct association between the PLAY system and psychological symptoms suggests that affective processes mediated by PLAY may function as distal or context-dependent influences rather than proximal predictors of psychopathology. PLAY may indirectly promote resilience through mechanisms such as social competence, stress recovery, and emotional regulation (Gray, 2011). Additionally, as PLAY behaviors are more prominent in childhood and adolescence and decline in adulthood, their association with clinical symptoms may be less detectable in cross-sectional studies (Pellis and Pellis, 2009; Vanderschuren et al., 1997).

A notable and unexpected finding revealed a direct negative association between the CARE system and depressive symptoms. This relationship may be elucidated through the interaction between the CARE and PANIC systems (Panksepp, 2010). According to Affective Neuroscience (AN) theory, reduced CARE activity combined with heightened PANIC activation generates a neural environment conducive to depressive states (Montag et al., 2021). In individuals with inherently low CARE system functioning, the buffering effects of social affiliation are weakened; when accompanied by PANIC system hyperactivation, this predisposes them to affective dysregulation and intensifies experiences of loss and emotional distress (Panksepp, 2010; Panksepp and Biven, 2012).

Our third hypothesis was supported. PANIC and FEAR were positively and indirectly associated with anxiety and depressive symptoms through increased attachment anxiety. These findings suggest that heightened activation of the FEAR and PANIC systems, in combination with intensified fears of abandonment and attachment insecurity, is associated with greater severity of anxiety and depressive symptoms (Dagan et al., 2018; Fuchshuber et al., 2024). As these systems regulate responses to threat and separation distress (Panksepp, 1998), their hyperactivation likely amplifies rejection sensitivity and reliance on attachment hyperactivation strategies, thereby exacerbating psychological symptoms (Mikulincer and Shaver, 2016).

Our fourth hypothesis—that CARE, PLAY, and SEEKING would be negatively and indirectly related to anxiety and depressive symptoms through increased connectedness to nature—was only partially supported, as this relationship emerged for CARE and SEEKING. These results suggest that the activation of the CARE and SEEKING systems may constitute an initial mechanism underlying individual differences in the psychological benefits derived from contact with nature (Ferrajão et al., 2024; Pihkala, 2020), a process that we refer to as the Biophilic Connection to Nature. Interaction with nature may engage the CARE system by promoting empathy and nurturing behaviors and stimulate the SEEKING system through exposure to novelty and complexity, thereby enhancing curiosity and attentional engagement (Kellert and Wilson, 1993; Panksepp and Biven, 2012). This interpretation aligns with the Biophilia Hypothesis, which posits an innate human tendency to seek connection with nature as a source of psychological wellbeing (Kellert and Wilson, 1993; Wilson, 1984).

Our fifth hypothesis, which proposed that higher levels of ANGER would indirectly predict increased anxiety and depressive symptoms through reduced connectedness to nature, was fully supported. These findings suggest an additional pathway—labeled the Anger–Nature Disconnection—that may account for individual differences in the psychological benefits derived from nature exposure. This pathway appears characteristic of individuals with heightened activation of the anger system, a temperamental trait associated with irritability and frustration proneness (Montag and Davis, 2018), which correlates negatively with connectedness to nature (Di Fabio and Kenny, 2021; Di Fabio and Rosen, 2019). Such diminished connectedness may represent a risk factor for psychological distress (Pouso et al., 2021).

Our serial mediation analyses revealed distinct pathways linking primary emotional systems to psychological symptoms via attachment patterns and connectedness to nature. Specifically, CARE, SEEK, and PLAY were positively associated with attachment dependence, which in turn were linked to greater connectedness to nature, subsequently associated with lower anxiety and depressive symptoms. These results suggest a distinct mechanism by which connectedness to nature influences psychological wellbeing, emphasizing the mediating role of attachment dependence rather than attachment closeness in this relationship.

Individuals exhibiting higher attachment dependence may demonstrate increased activation of positive emotional systems–CARE, PLAY, and SEEKING-supporting social bonding, exploration, and affiliative behaviors (Panksepp, 1998; Panksepp and Biven, 2012). Such dependence—marked by the pursuit of reassurance within secure relationships—can foster a secure attachment orientation that enhances safety, curiosity, and connection with the natural environment (Lumber et al., 2017; Weinstein et al., 2009). In this sense, nature may function as an extension of the attachment system by providing experiences of safety, emotional regulation, and restoration analogous to those associated with a secure attachment figure (Jordan, 2009; Little and Derr, 2018). Repeated positive interactions with natural environments may therefore reinforce a stable sense of connectedness to nature, conceptualized as Attachment to Nature, in which nature serves as a psychologically secure base that supports affect regulation, curiosity, resilience, and psychological wellbeing (Lumber et al., 2017; Scannell and Gifford, 2017).

Finally, two distinct paths emerged linking the PANIC and FEAR systems to psychological symptoms via attachment orientations and connectedness to nature. PANIC and FEAR were positively associated with attachment anxiety, which in turn were linked to heightened anxiety and depressive symptoms, while attachment anxiety showed no relation to connectedness to nature. Unexpectedly, PANIC and FEAR were negatively associated with attachment dependence, which was linked to lower connectedness to nature and, consequently, to greater anxiety and depressive symptom severity.

These findings identify a fourth pathway through which connectedness to nature influences psychological wellbeing, mediated by the FEAR and PANIC systems, herein termed Fear-Based Nature Disconnection. This pathway involves two routes linking attachment and nature connectedness to psychological symptoms. Specifically, heightened FEAR and PANIC system activation, coupled with elevated attachment anxiety, appears to foster a psychological disconnection from nature. Such individuals exhibit increased sensitivity to perceived threats and intense distress in response to social separation, which may hinder experiences of nature as safe, restorative, or affiliative (Panksepp, 1998; Panksepp and Biven, 2012; Little and Derr, 2018). Chronic overactivation of these systems may lead to perceptions of the natural environment as unpredictable or hostile, especially under conditions of solitude or low control (Mayer et al., 2009; Mikulincer and Shaver, 2016).

Individuals with hyperactive FEAR and PANIC systems, who are uncomfortable relying on others, tend to exhibit lower connectedness to nature. These temperamental traits, characterized by autonomy, emotional distance, and reduced relational trust (Bartholomew and Horowitz, 1991), may hinder affective and experiential engagement with the natural world (Mayer and Frantz, 2004; Tam, 2013). Consequently, such individuals may perceive nature as less safe or emotionally significant, limiting the formation of an affective bond and the associated psychological benefits (Little and Derr, 2018). This Fear-Based Nature Disconnection pathway may thus constrain restorative experiences in natural environments.

**Figure 2:**
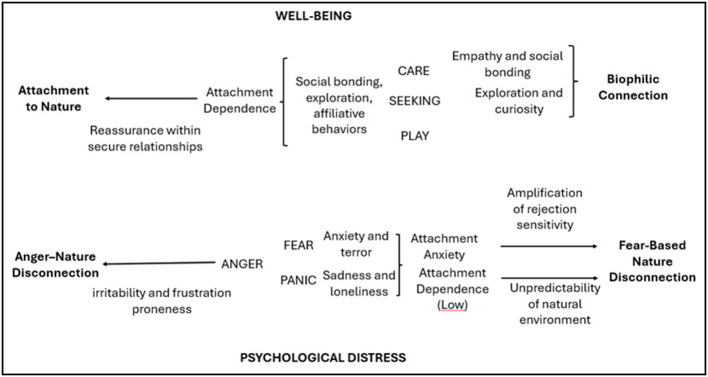
Pathways from Nature Connectedness to Psychological WellBeing. Individual differences in nature connectedness associated with psychological wellbeing, through primary emotional systems and attachment patterns. This figure illustrates the proposed model in which individual differences in nature connectedness modulate the association between nature exposure and psychological wellbeing. The relationship is explained by the mediating role of primary emotional systems (CARE, PLAY, SEEKING, ANGER, FEAR, PANIC) and attachment patterns (dependence and anxiety).

Our findings indicate that individual connectedness to nature is linked to either resilience against or susceptibility to anxiety and depression, highlighting its role in explaining variability in the mental health benefits of nature exposure. Moreover, the results provide preliminary empirical support for four pathways through which connectedness to nature may influence psychological wellbeing: biophilic connection, attachment to nature, anger–nature disconnection, and fear-based nature disconnection (Figure 2).

The findings indicate that temperamental traits marked by elevated positive emotional systems (CARE, PLAY, SEEKING) and secure attachment—particularly comfort with dependence—promote a strong connection to nature (Biophilic Connection to Nature and Attachment to Nature), which appear to be protective against psychological symptoms. In contrast, traits associated with negative emotional systems (ANGER, FEAR, PANIC) and insecure attachment correspond to weaker or disconnected relationships with nature (Anger–Nature Disconnection and Fear-Based Nature Disconnection), potentially increasing vulnerability to anxiety and depression.

## 5 Limitations and future research

The present findings should be interpreted in light of several limitations. The sample was not representative, limiting generalizability; future research should employ probabilistic sampling. The cross-sectional design precludes causal inference, warranting longitudinal studies to clarify temporal and causal relationships. The sample was recruited through convenience sampling via online platforms and social media, which may have introduced self-selection bias. Individuals with stronger interests in mental health, emotional functioning, or nature-related topics may have been more likely to participate. Although the sample was substantial, the overrepresentation of women may limit the generalizability of the findings, particularly given documented gender differences in ~primary emotional systems (Fuchshuber et al., 2019b), attachment orientations (Del Giudice, 2011), and connectedness to nature (Di Fabio and Rosen, 2019). Consequently, the observed associations may predominantly reflect patterns characteristic of female participants. Future studies should replicate these findings using more demographically balanced samples and examine potential gender-specific pathways linking emotional systems, attachment processes, connectedness to nature, and psychological wellbeing. Additionally, participants' past or current exposure to natural environments was not assessed, though such contact may influence primary emotional systems, nature connectedness, and psychological symptoms; future studies should include measures of environmental exposure. Examining these relationships across diverse contexts would further strengthen ecological validity and generalizability.

Despite these limitations, the findings have implications for clinical practice and nature-based interventions, suggesting that approaches such as ecotherapy and mindful engagement with nature may support mental health and psychological resilience (Capaldi et al., 2017). Integrating such interventions into therapeutic settings may strengthen individuals' connectedness to nature, particularly when aligned with attachment processes and emotion regulation. Targeting positive emotional systems while modulating negative systems may further enhance biophilic and attachment-related mechanisms. Clinically, interventions may be tailored to primary emotional profiles: individuals with elevated FEAR or PANIC may benefit from structured, safe, and predictable nature exposure (e.g., guided walks in familiar environments); those with reduced SEEKING may respond more favorably to exploratory and novelty-based activities designed to increase curiosity, motivation, and behavioral engagement; and individuals with elevated ANGER may benefit from mindfulness-based nature interventions aimed at improving emotional regulation and attentional control (Buzzell and Chalquist, 2009; Jordan and Hinds, 2016).

Future research should employ longitudinal and experimental designs to examine how temperamental traits, attachment, and nature connectedness affect psychological outcomes. The four proposed pathways—Biophilic Connection, Attachment to Nature, Anger–Nature Disconnection, and Fear-Based Nature Disconnection—warrant cross-cultural validation. Exploring physiological and neurobiological mechanisms, alongside moderators such as personality, environmental attitudes, and urbanization, may clarify how nature contact facilitates emotional regulation and wellbeing.

## Data Availability

The raw data supporting the conclusions of this article will be made available by the authors, without undue reservation.
